# The role of environmental and healthcare-associated infections in Asia: Lessons learned from the coronavirus disease 2019 (COVID-19) pandemic

**DOI:** 10.1017/ash.2023.182

**Published:** 2023-06-15

**Authors:** Anucha Apisarnthanarak, Moi Lin Ling, David J. Weber

**Affiliations:** 1 Department of Internal Medicine, Faculty of Medicine, Thammasat University, Pathum Thani, Thailand; 2 Infection Prevention & Epidemiology, Singapore General Hospital, Outram Road, Singapore, Singapore; 3 Gillings School of Global Public Health, University of North Carolina, Chapel Hill, North Carolina, United States

Environmental cleaning and disinfection practices have been shown to reduce microorganism bioburden in the healthcare environment.^1^ Due to the coadministration of high-dose steroids and the prolonged duration of mechanical ventilation, coinfection with multidrug-resistant organisms (MDROs) among coronavirus disease 2019 (COVID-19) patients is common in critical care patients, with a prolonged length of stay in critical care units.^2^ Furthermore, the rush of creating of negative-pressure units during the COVID-19 pandemic may have resulted in some outbreaks associated with molds such as *Aspergillus* and *Zygomyces*.^3^ In countries where they are prevalent, emerging fungal outbreaks, such as *Candida auris* outbreaks, have been reported.^4^ The control of MDROs among COVID-19 patients is also difficult, given the requirement for airborne plus contact isolation among these patients and the difficulty in wearing and changing personal protective equipment (PPE) in a critical care unit.^2^ The control of invasive fungal infections (IFIs) also requires practice of proper infection prevention and control (IPC) during construction and renovation.^3^ In this commentary, we provide evidence of how environment contribute to HAIs in Asia. We discuss how to properly disinfect and decontaminate healthcare environment, and we provide suggestions on how to improve air quality and water quality during COVID-19 pandemics.

## How environment contributed to HAIs during the COVID-19 pandemic in Asia

It is well recognized that MDROs are selected by use of broad-spectrum antibiotics and often have an environmental reservoir that can facilitate rapid spread in critical care units if appropriate interventions are not introduced.^1^ Also, the constant use of gloves and gowns during a severe acute respiratory syndrome (SARS) outbreak led to an increase in transmission of MDROs, particularly methicillin-resistant *Staphylococcus aureus*.^5^ Outbreaks of MDROs can be more difficult to control in middle- and lower-income countries in Asia where infrastructure may not be adequate (eg, suboptimal design of negative-pressure airborne isolation units and inadequate nurse-to-patient ratios).^2^ During the COVID-19 pandemic, airborne transmission of some MDR gram-negative pathogens, such as *Acinetobacter baumannii*, has also been reported.^6^ Evidence of airborne transmission of MDR-*A. baumannii* supports the view that placing patients in cohorts in enclosed cubicles with partitions and a closed door is preferred if single rooms are not available.^6^ Such conditions require a practical strategy to control MDROs in resource-limited settings. Additional strategies are also needed in situations that do not allow PPE changing easily between cases in the cohort areas. These strategies must feature multimodal approaches that include risk stratification for index patients that may potentially harbor MDROs with isolation in single rooms, an antimicrobial stewardship program for COVID-19 patients, and policies to discontinue COVID-19 precautions as well as a policy to perform robust terminal environmental disinfection.^2^ Such strategies, together with full compliance with infection prevention measures, will help limit the transmission of MDROs in COVID-19 cohort areas in critical care units. Antimicrobial stewardship strategies, including inappropriate antibiotic use featuring the combination of procalcitonin and clinical pulmonary infection score (CPIS), have been associated with successfully reducing inappropriate antibiotic use among severely to critically ill COVID-19 patients, for multidrug-resistant organisms, and for invasive fungal infections during the intervention.^7,8^

Likewise, hospital construction and renovations activities are never ending, and they are the main cause of healthcare-associated fungal outbreaks including Aspergillus and Zygomycetes,^9^ which pose serious threats to immunocompromised hosts, including high-risk patients (eg, COVID-19 pneumonia patients). Implementing an infection control risk assessment (ICRA) for renovation and construction decreases the risk of healthcare-associated fungal outbreaks. However, ICRAs are not typically performed in developing countries, leading to healthcare-associated fungal outbreaks in settings such as ICUs, hematologic wards, and renal transplant units.^9^ Although uncommon, healthcare-associated fungal outbreaks must be suspected when 2 or more cases occur over a short period.^3^ Early outbreak investigation and identification of the source are likely to play a key role in limiting the spread of healthcare-associated fungal outbreaks. Such strategies, together with a full compliance with recommendations from a multidisciplinary coordination team, will help limit the construction-related fungal infection especially in a COVID-19 ICU.

## Selected emerging infectious diseases and germicide susceptibility

SARS-CoV-2 can survive on surfaces for minutes to hours, but contaminated surfaces have only rarely been linked to transmission.^10^ Contaminated elevator buttons were hypothesized via indirect transmission to lead to outbreaks in a mall and in an apartment building. SARS-CoV-2 has been demonstrated in multiple studies to survive on a variety of surfaces (ie, steel, plastics, paper, disposable hospital gowns, wood, glass) for minutes to days; survival was better at low temperature and humidity. SARS-CoV-2 RNA has been detected in a mean of ∼15% of hospital room samples. Despite the frequent finding of SARS-CoV-2 contamination, viable virus was not commonly detected.^10^ SARS-CoV-2 is susceptible to alcohol hand disinfectant agents (ie, ethanol, 60%–90%; isopropanol, ∼75%), which are rapidly effective (ie, 15 seconds) in eliminating >3-log_10_ SARS-CoV-2 from skin. Povidone-iodine (0.23%-7.5%) is also effective in inactivating SARS-CoV-2 strains in 15 seconds. SARS-CoV-2 is inactivated by sodium hypochlorite and by hydrogen peroxide (0.5%) in 1 minute. SARS-CoV-2 is also inactivated by quaternary ammonium compounds in 30 seconds to several minutes. Disinfectant cloths are also effective in eliminating SARS-CoV-2 in 2 minutes. The environmental survival and germicide susceptibilities of SARS-CoV-2, Middle Eastern respiratory syndrome coronavirus, and Ebola virus have been reviewed.^11^ All 3 viruses may survive for hours on environmental surfaces, and because all are enveloped viruses, they are susceptible to standard antiseptics (eg, alcohol) and disinfectants (eg, hypochlorite). Specific germicides that have demonstrated effectiveness against coronavirus are listed by the US Environmental Protection Agency and against Ebola.^11^

Selecting appropriate disinfectant is key to effective healthcare environment disinfection. Some emerging infectious diseases such as *Candida auris* and monkeypox (mpox) may require further understanding on principle of disinfection in healthcare environment as well as more evidence-based on the proper disinfectant. *C. auris* is most commonly transmitted via direct contact (patient to patient) and indirect contact (contaminated environment such as sharing the same hospital room or being admitted to a room previously occupied by a MDRO colonized or infected patient).^11^ Widespread contamination of the surface environment has been reported. In laboratory tests, *C auris* has been demonstrated to persist for days on environmental surfaces and linens. *C. auris* is susceptible to sterilants and chemicals used for high-level disinfectants.^11,12^ Effective low-level disinfectants (used to decontaminate environmental surfaces) have included 70% isopropyl alcohol, 1:10 dilution of 5.25% sodium hypochlorite, 0.5% to 1.4% hydrogen, 0.5% quaternary ammonium compound, and 55% isopropyl alcohol and other products. Alcohols are the antiseptic of choice for hand hygiene based on *in vitro* testing. The US EPA has published a list of germicides active against *C. auris*.^11,12^ For mpox, environmental contamination in household or hospital rooms occupied by patients with mpox as assessed by PCR was common, but demonstration of viable virus was less common. There are no published studies of the survival of mpox virus, but based on studies of other pox viruses (eg, variola, vaccinia), prolonged survival is expected (ie, weeks to months). Similarly, there are no published studies on the susceptibility of mpox virus to disinfectants, but based on studies of other pox viruses, mpox virus is likely susceptible to sodium hypochlorite (0.636% active chlorine), quaternary ammonium combined with chlorhexidine, and quaternary ammonium combined with glutaraldehyde. Ethanol (50%–70%) and isopropyl (40%–50%) are likely effective with a 1-minute contact time.

## Improvement of air and water quality in Asia Pacific: What is needed?

### Air quality

One of the lessons learned during the COVID-19 pandemic was the important role ventilation plays in the transmission of the virus in healthcare setting. The outbreaks reported among staff, patients, and visitors to the healthcare facility^13,14^ highlighted that attention needs to be given to adequate air flow and correct directional flow. The World Health Organization guidance on addressing the inadequacy was a timely help for many during the crisis.^15^ There is an urgent call for all healthcare institutions to review the ventilation in all areas, even for daily patient care, because it is fundamental to ensure adequate ventilation to prevent healthcare-associated infections associated with common respiratory viral pathogens such as influenza, respiratory syncytial virus, etc. This review work is best conducted in consultation with the facility engineers and IPC professionals as a team. Inappropriate design of the healthcare facility may often be a root cause for inadequate ventilation. Although there may be specifications for the design in some developed countries, these may be absent in low- and middle-income countries. Nevertheless, it would be expected that a healthcare facility be designed to facilitate safe patient care. The involvement of IPC from the early phase of planning until end of the project is recommended. Provision for more negative-pressure facilities may be made as part of pandemic preparedness. These may either be the installation of portable negative pressure anterooms to existing rooms or the construction of new negative-pressure isolation rooms built according to specifications. Adjunct measures may be considered such as the use of UV-C technology in room decontamination.

### Water quality

Reports on gram-negative bacterial outbreaks in association with biofilms in sinks and drains have alerted us to potential reservoirs in the patient care areas that demand closer attention to use and maintenance of the water system in healthcare facilities.^16^ Water stagnation has its associated risks of *Legionella* growth in water system as reported by some during building closure during the COVID-19 pandemic or renovation.^17^ A water management program with the involvement of a multidisciplinary team including the IP professional, is an essential component in the IPC program to address this. The provision of safe water for drinking and keeping biofilms away from the water plumbing system are issues that this program can focus on as a start.

In conclusion, ensuring a clean and hygienic environment in the healthcare facility is one of the core components of an IPC program. This goes beyond environmental cleaning and requires the IPC professional to collaborate with environmental services and facility management colleagues in achieving good indoor air and water quality. The Asia Pacific Society of Infection Control (APSIC) will work to strengthen the evidence on healthcare environmental cleaning and disinfection and will develop strategies to disseminate best practices throughout Asia Pacific in the years to come.
